# Updates in Rhea—a manually curated resource of biochemical reactions

**DOI:** 10.1093/nar/gku961

**Published:** 2014-10-20

**Authors:** Anne Morgat, Kristian B. Axelsen, Thierry Lombardot, Rafael Alcántara, Lucila Aimo, Mohamed Zerara, Anne Niknejad, Eugeni Belda, Nevila Hyka-Nouspikel, Elisabeth Coudert, Nicole Redaschi, Lydie Bougueleret, Christoph Steinbeck, Ioannis Xenarios, Alan Bridge

**Affiliations:** 1 Swiss-Prot Group, SIB Swiss Institute of Bioinformatics, Geneva, CH-1206, Switzerland; 2 Equipe BAMBOO, INRIA Grenoble Rhône-Alpes, Montbonnot Saint-Martin, F-38330, France; 3 Cheminformatics and Metabolism Team, European Bioinformatics Institute, Hinxton, CB10 1SD, UK; 4 Vital-IT, SIB Swiss Institute of Bioinformatics, Lausanne, CH-1015, Switzerland; 5 Genoscope—LABGeM, CEA, Evry, F-91057, France; 6 Department of Biochemistry, University of Geneva, Geneva, CH-1206, Switzerland

## Abstract

Rhea (http://www.ebi.ac.uk/rhea) is a comprehensive and non-redundant resource of expert-curated biochemical reactions described using species from the ChEBI (Chemical Entities of Biological Interest) ontology of small molecules. Rhea has been designed for the functional annotation of enzymes and the description of genome-scale metabolic networks, providing stoichiometrically balanced enzyme-catalyzed reactions (covering the IUBMB Enzyme Nomenclature list and additional reactions), transport reactions and spontaneously occurring reactions. Rhea reactions are extensively curated with links to source literature and are mapped to other publicly available enzyme and pathway databases such as Reactome, BioCyc, KEGG and UniPathway, through manual curation and computational methods. Here we describe developments in Rhea since our last report in the 2012 database issue of Nucleic Acids Research. These include significant growth in the number of Rhea reactions and the inclusion of reactions involving complex macromolecules such as proteins, nucleic acids and other polymers that lie outside the scope of ChEBI. Together these developments will significantly increase the utility of Rhea as a tool for the description, analysis and reconciliation of genome-scale metabolic models.

## AIMS AND SCOPE OF RHEA

**Figure 1.**
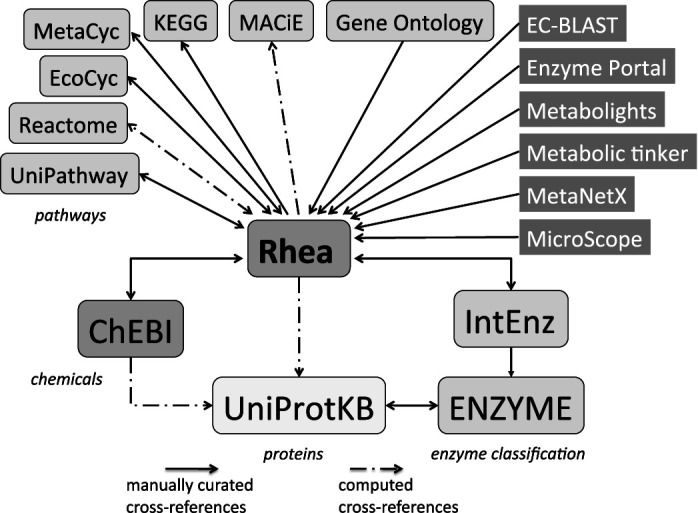
Interactions between Rhea and other resources. Rhea provides cross-references to chemical entities of ChEBI (2), to biochemical reactions of EcoCyc (19), MetaCyc (20), KEGG (18), MACiE (16), Reactome (17) and UniPathway (21), to EC numbers of IntEnz (10) and to protein sequences of UniProtKB (15). The Gene Ontology (GO) is closely aligned with ChEBI , and GO molecular functions describing enzymatic reactions cross-reference Rhea (29). Rhea is one of the reaction repositories employed by MicroScope (9), an integrated resource for the curation and comparative analysis of genomic and metabolic data of microbes. Rhea also provides metabolic reactions for a number of other resources including the EBI Enzyme Portal (5), the reference layer of the MetaboLights resource (6), the metabolic model analysis and reconciliation platform of MetaNetX.org (7,8), EC-BLAST (11) and Metabolic tinker (12).

Rhea is a manually curated resource of biochemical reactions for the functional annotation of enzymes and the description of genome-scale metabolic networks (1). Rhea provides stoichiometrically balanced descriptions for enzyme-catalyzed reactions, transport reactions and spontaneously occurring reactions using chemical species from the Chemical Entities of Biological Interest (ChEBI) ontology (2), specifying reaction constituents, their stoichiometric coefficients and relative locations. This information is manually curated from peer-reviewed literature by experts. Each Rhea reaction is assigned a unique identifier, with uniqueness ensured by the calculation of a fingerprint for each reaction which considers the constituent compounds, their stoichiometry and localization. Reaction constituents are represented by the major micro-species at pH 7.3 (verified using the Marvin pKa calculator from ChemAxon (version 6.2.0, http://www.chemaxon.com)). All reactions are stoichiometrically balanced for both mass and charge, which facilitates the use of Rhea for the construction, analysis, comparison and reconciliation of genome-scale metabolic models (3,4). More details on the representation of reactions can be found in our preceding paper (1). Rhea provides metabolic reactions for a number of other biological data and knowledge resources including the EBI Enzyme Portal (5), the reference layer of the MetaboLights resource (6), the metabolic model analysis and reconciliation platform of MetaNetX.org (7,8), the microbial genomic annotation platform MicroScope (9) and IntEnz, a reference for the recommendations of the Nomenclature Committee of the International Union of Biochemistry and Molecular Biology (NC-IUBMB) on the nomenclature and classification of enzymes (10). Rhea reactions are also used by tools such as EC-BLAST, a tool to automatically search and compare biochemical reactions (11) as well as Metabolic tinker (12), an online tool for guiding the design of synthetic metabolic pathways. Interactions between Rhea and other resources are described in Figure 1.

## EXTENDING RHEA TO COMPLEX MACROMOLECULES AND POLYMERS

Computational models of cellular metabolism generally include instances of complex biological macromolecules such as proteins, nucleic acids and other polymers that lie outside the scope of ChEBI, which deals with small molecules and metabolites. To permit the representation of reactions involving such entities in Rhea, we have introduced generic compounds (‘Rhea generics’) and polymers (‘Rhea polymers’). Rhea generics represent complex biological macromolecules such as proteins, nucleic acids and complex polysaccharides. Rhea polymers represent compounds that appear on both sides of a given reaction with different relative polymerization indices, such as ‘*n*’ and ‘*n* + 1’.

**Figure 2.**
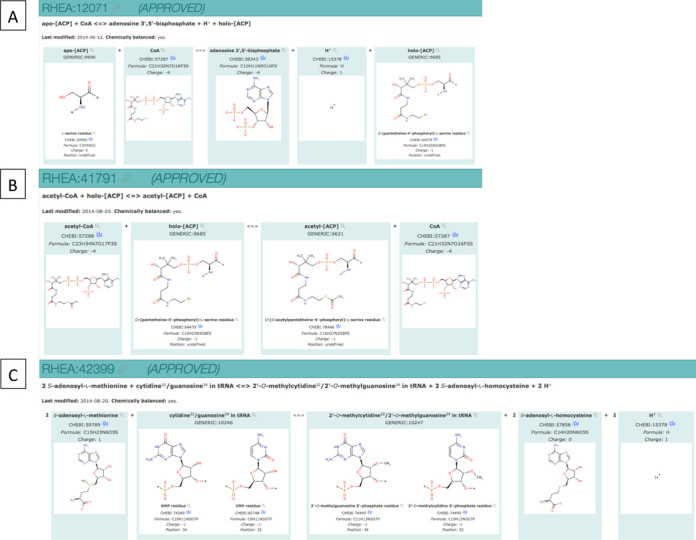
Rhea generics. (**A**) RHEA:12071 represents the priming of acyl carrier protein (ACP) by ACP synthase, where a phosphopantetheine group from coenzyme A (CoA) is attached to a conserved serine residue of ACP to form the activated holo-[ACP] and to release adenosine 3′,5′ bisphosphate (CHEBI:58343). Apo-[ACP] is modeled by a single l-serine residue (GENERIC:9690) and holo-[ACP] by an *O*-(pantetheine-4′-phosphoryl)-l-serine residue (GENERIC:9685). (**B**) RHEA:41791 represents the initiation of fatty acid synthesis on holo-[ACP] in which an acetyl group is transferred from acetyl CoA (CHEBI:57288) to the *O*-(pantetheine-4′-phosphoryl)-l-serine residue of the holo-[ACP] (GENERIC:9685), forming acetyl-[ACP] (GENERIC:9621) that includes an *O*-(*S*-acetylpantetheine-4′-phosphoryl)-l-serine residue (CHEBI:78446). (**C**) RHEA:42399 illustrates the use of Rhea generics composed of multiple residues. The substrate is Rhea GENERIC:10246, which includes both a CMP residue (CHEBI:82748) at position 32 and a GMP residue (CHEBI:74269) at position 34. The corresponding product is Rhea GENERIC:10247, which carries the corresponding 2′-*O*-methylcytidine 5′-phosphate residue (CHEBI:74495) and 2′-*O*-methylguanosine 5′-phosphate residue (CHEBI:74445) at the same positions.

We describe first the use of Rhea generics. Each Rhea generic has a unique identifier and a name that specifies the nature of the biological macromolecule under consideration. Residues and functional groups that are modified during the course of the reaction are represented explicitly using entities from ChEBI, which allows stoichiometric balancing for mass and charge. An example of the usage of Rhea generics is modification reactions involving acyl carrier protein (ACP), which plays an essential role in the process of fatty acid biosynthesis. Before ACP can accept acyl chains for elongation the protein must be activated by ACP synthase, which attaches a phosphopantetheine group from coenzyme A (CoA) to a conserved serine residue of ACP, releasing adenosine 3',5' bisphosphate (13). In Rhea, this post-translational modification is described by RHEA:12071 (Figure 2A). The substrate for this reaction is the Rhea generic ‘apo-[ACP]’ (GENERIC:9690), in which the target serine residue is represented by CHEBI:29999 (‘l-serine residue’). The product of this reaction is holo-[ACP] (GENERIC:9685), which includes an *O*-(pantetheine-4′-phosphoryl)-l-serine residue represented by CHEBI:64479. The initiation of fatty acid synthesis on holo-[ACP] is represented by RHEA:41791, in which an acetyl group is transferred from acetyl CoA (CHEBI:57288) to the *O*-(pantetheine-4′-phosphoryl)-l-serine residue of the holo-[ACP] (GENERIC:9685), forming acetyl-[ACP] (GENERIC:9621) that includes an *O*-(*S*-acetylpantetheine-4′-phosphoryl)-l-serine residue (CHEBI:78446) (Figure 2B). Note that the same residue or group may appear in a number of distinct generic compounds and reactions (such as the l-serine residue CHEBI:29999). In some reactions several residues and/or functional groups from a single Rhea generic macromolecule may participate in the same chemical transformation. The yeast enzyme tRNA (cytidine^32^/guanosine^34^−2′-*O*)-methyltransferase (described in UniProtKB/Swiss-Prot record P38238) catalyzes the formation of 2′-*O*-methylribose at two sites in the anticodon loop of a single tRNA molecule (14). This reaction is RHEA:42399 (Figure 2C). Its substrate is Rhea GENERIC:10246, which includes both a cytidine 5′-phosphate residue (CHEBI:82748) at position 32 and a guanosine 5′-phosphate residue (CHEBI:74269) at position 34. The corresponding product is Rhea GENERIC:10247, which carries the corresponding 2′-*O*-methylcytidine 5′-phosphate residue (CHEBI:74495) and 2′-*O*-methylguanosine 5′-phosphate residue (CHEBI:74445) at the same positions.

Rhea polymers differ from Rhea generics. Rhea polymers have been introduced in order to allow balancing of polymerization reactions that include different abstract polymerization indices for polymers such as ‘*n*’ and ‘*n* + 1’, as in this example:

\begin{eqnarray*} &&{\rm GDP}{-}\alpha{-}{\rm {\small d}{-}glucose}{+}(1,4{-}\beta {\rm{-}{\small d}{-}glucosyl)}_{\rm n} \\ &&= {\rm GDP}{+}{\rm (1,4 - }\beta {\rm{-}{\small d}{-}glucosyl)}_{{\rm n} + 1} \end{eqnarray*}

**Figure 3.**
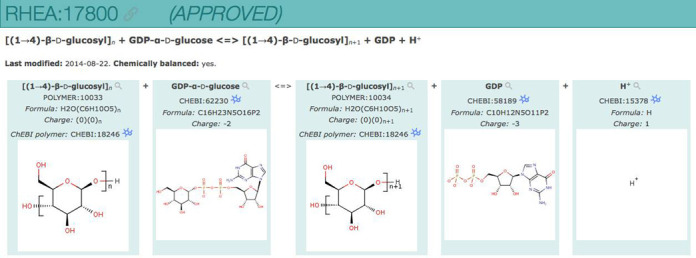
Rhea polymers. RHEA:17800 is a polymerization reaction where the glucosyl moiety of GDP-α-d-glucose (CHEBI:62230) is added to (1,4-β-d-glucosyl)*_n_* (POLYMER:10033) through a β-1→4 linkage to produce (1,4-β-d-glucosyl)*_n_*_+1_ (POLYMER:10034).

ChEBI contains only a single Instance of each abstract polymer, with a single unknown polymerization index. Each Rhea polymer has an identifier (prefixed by ‘POLYMER’), a name, a link to the corresponding ChEBI polymer and a relative polymerization index. Several Rhea polymers may share the same ChEBI entry, but they must have different polymerization indices, which are used in reaction balancing. The use of Rhea polymers in the context of polymerization reactions is shown in Figure 3.

## RHEA CONTENT

Rhea has grown steadily since our last report through the expert curation of new chemical entities and reactions from peer-reviewed literature. At the time of writing, Rhea (release 53) includes 7044 unique reactions involving 5927 unique reaction participants, and cites 2766 unique PubMed identifiers (see http://www.ebi.ac.uk/rhea/statistics.xhtml for details). This corresponds to a 63% increase in the number of unique reactions and a 161% increase in the number of unique citations since our last publication in 2012 (Rhea release 24, containing 4321 unique reactions, 3788 unique reaction participants and citing 1058 unique PubMed identifiers).

The value and utility of Rhea reactions are enhanced by extensive cross-references to other public resources (Table 1). The cross-references are extensively manually curated and crosschecked, with information on possible corrections and clarifications being regularly exchanged between curators of Rhea and those of the other resources. In addition, cross-references are automatically added from Rhea to UniProtKB/Swiss-Prot (15) protein records (through Enzyme Commission (EC) numbers in IntEnz) and to reaction descriptions in MACiE (16) and Reactome (17) (through shared participants).

**Table 1.** Cross-references in Rhea (release 53)

Database	Ref.	Data type	Origin	Total	Unique
ChEBI http://www.ebi.ac.uk/chebi	(2)	Compound	Manually curated	36 928	6720
IntEnz http://www.ebi.ac.uk/intenz	(10)	EC number	Manually curated	4955	4110
MetaCyc http://metacyc.org	(20)	Reaction	Manually curated	4407	4279
EcoCyc http://ecocyc.org	(19)	Reaction	Manually curated	1136	1109
KEGG http://www.genome.jp/kegg	(18)	Reaction	Manually curated	3724	3641
UniPathway http://www.unipathway.org/pathway	(21)	Reaction	Manually curated	1878	1835
Reactome http://www.reactome.org	(17)	Reaction	Automatic via ChEBI IDs	608	582
UniProtKB www.uniprot.org	(15)	Protein	Automatic via EC numbers	22 0058	16 1289
PubMed http://www.ncbi.nlm.nih.gov/pubmed		Bibliographic citation	Manually curated	5164	2766

## SUBMISSIONS TO RHEA

Rhea welcomes submissions describing new reactions or suggesting updates to existing reactions. All submissions should be posted on our SourceForge Reaction Requests/Updates tracker (http://sourceforge.net/p/rhea-ebi/reaction-requests-updates) with relevant information (name, 2D structure…) for each reaction participant and cross-references to other relevant databases and source literature where available. Reactions requested for a publication under review are assigned preliminary status during the peer-review process and acquire approved status once the manuscript has been accepted.

## RHEA AVAILABILITY

The Rhea web server (http://www.ebi.ac.uk/rhea) provides programmatic access as well as browsing, searching and download facilities. Details of common search options — including compound names, compound and reaction identifiers, reaction equations, EC numbers, UniProtKB/Swiss-Prot accession numbers, bibliographic citations and identifiers from external cross-referenced resources such as KEGG (18), EcoCyc (19), MetaCyc (20), UniPathway (21), MACiE or Reactome — are provided in our last publication (1). Searches with compound identifiers may be prefixed with CHEBI, POLYMER or GENERIC to specify the desired type of molecule. Rhea generics and polymers may also be retrieved by searching for the associated ChEBI residue/group or compound (e.g. ‘CHEBI:29999’) or by name (e.g. ‘ACP’). It is possible to link to reactions in the public web site using the following URL template http://www.ebi.ac.uk/rhea/reaction.xhtml?id=, adding the numerical reaction identifier as in this example: http://www.ebi.ac.uk/rhea/reaction.xhtml?id=10499.

All Rhea data is available for free download (http://www.ebi.ac.uk/rhea/download.xhtml) in BioPAX level 2 (biopax2) (22), RXN and RD (23) formats. In the BioPAX level 2 distribution of Rhea all reaction participants are defined by the class ‘physicalEntityParticipant’. Cross-references to other databases such as ChEBI, EcoCyc, IntEnz, KEGG, MACiE, MetaCyc, Reactome, UniPathway and UniProtKB are also available as tab-separated text files. The 2D structures of chemical compounds used in Rhea are available for download either as individual molfiles or as a Structure-Data File (SDF). These chemical formats are specified by Accelrys (formerly by Molecular Design Limited (MDL) (23)).

Rhea RESTful web services allow reactions to be retrieved in BioPAX level 2 (22), CMLReact (cmlreact) (24) or RXN CTfile (rxn) (23) formats by querying for their identifier or other terms. Example queries are provided in the online documentation. Rhea also provides a BioJS component (BioJS.Rheaction, http://www.ebi.ac.uk/Tools/biojs/registry/Biojs.Rheaction.html), which can be used to display (and possibly modify the layout of) a Rhea reaction in an external web page given only its Rhea ID. The EBI Enzyme Portal makes use of this BioJS component to display reactions (example: http://www.ebi.ac.uk/enzymeportal/search/P45850/reactionsPathways).

## FUTURE DIRECTIONS

We are actively developing Rhea as a vocabulary for the functional annotation of enzymes in UniProtKB. This annotation is currently provided using the enzyme classification (EC numbers) of the Enzyme Nomenclature committee of the IUBMB and textual reaction descriptions sourced from the ENZYME database (25) (itself derived from IntEnz). Our current work involves the translation of all outstanding IUBMB reactions (the majority of which involve generic compounds or polymers) into Rhea. We are also expanding Rhea to cover the hundreds of enzyme activities that are not yet described by the IUBMB classification (26–28), many of which already have textual reaction descriptions annotated in UniProtKB (one example being the aforementioned GDP-α-d-mannose hydrolysis reaction RHEA:28105 described in UniProtKB/Swiss-Prot record P32056). We will also exploit the underlying ontology of ChEBI in order to provide a logical reaction classification based on the curated relations between reaction participants. This will serve as a useful complement to the classification of enzymatic activities by the IUBMB.
